# Pharmacological mechanisms of sodium-glucose co-transporter 2 inhibitors in heart failure with preserved ejection fraction

**DOI:** 10.1186/s12872-022-02693-8

**Published:** 2022-06-10

**Authors:** Bo Liang, Yi Liang, Ning Gu

**Affiliations:** 1 Nanjing University of Chinese Medicine, Nanjing, China; 2 Department of Cardiology, The Second Affiliated Hospital, School of Medicine, Zhejiang University, Hangzhou, China; 3 Southwest Medical University, Luzhou, China; 4 Nanjing Hospital of Chinese Medicine Affiliated to Nanjing University of Chinese Medicine, Nanjing, China

**Keywords:** Sodium-glucose co-transporter 2 inhibitors, Heart failure with preserved ejection fraction, Network pharmacology, Virtual screening, Molecular docking

## Abstract

**Background:**

More and more evidence indicates sodium-glucose co-transporter 2 inhibitors (SGLT2is) may display clinical benefits for heart failure with preserved ejection fraction (HFpEF). However, the mechanisms of the action remain unclear.

**Methods:**

A systematic pharmacology-based strategy was applied for predicting the potential molecular mechanisms of SGLT2is in HFpEF. The potential targets of SGLT2is and HFpEF were contained from diverse databases. After networks were constructed, Metascape was applied to functional enrichment. Moreover, the key findings were validated through molecular docking.

**Results:**

We obtained 487 SGLT2is related targets and 1505 HFpEF related targets. The networks showed the complex relationship of HFpEF-target-HFpEF. The results of functional enrichment analysis suggested that several biological processes, including muscle system process, inflammatory response, vasculature development, heart development, regulation of MAPK cascade, positive regulation of ion transport, negative regulation of cell population proliferation, cellular response to nitrogen compound, apoptotic signaling pathway, multicellular organismal homeostasis, response to oxidative stress, regulation of cell adhesion, positive regulation of cell death, response to growth factor, and cellular response to lipid, and signaling pathways, such as cardiomyopathy, cAMP signaling pathway, cytokine-cytokine receptor interaction, apoptosis, MAPK signaling pathway, HIF-1 signaling pathway, calcium signaling pathway, and NF-kappa B signaling pathway. Finally, we validated the interactions and combinations of SGLT2is and core targets.

**Conclusion:**

SGLT2is play the potential role of anti-HFpEF through the direct or indirect synergy of multiple targets and pathways. Our study promotes the explanation of the molecular mechanisms of SGLT2is in HFpEF.

**Supplementary Information:**

The online version contains supplementary material available at 10.1186/s12872-022-02693-8.

## Introduction

Heart failure (HF) is a complex clinical syndrome that results from any structural or functional impairment of ventricular filling or ejection of blood [1]. Ejection fraction (EF) is considered important in the classification of patients with HF [2] because of differing patient demographics, comorbid conditions, prognosis, and response to therapies [3] and because most clinical trials selected patients based on EF [4]. In the latest American and European guidelines, HF with preserved EF (HFpEF, EF ≥ 50%) was proposed [5, 6]. In patients with clinical HF, studies estimate that the prevalence of HFpEF is approximately 50% (range from 40 to 71%) [7]. Despite aggressive treatment, the residual risk of HFpEF remains high [8]. We have reason to believe that treatment strategies need to be continuously optimized and improved [9].

In recent years, more and more evidence shows that hypoglycemic drugs also have cardiovascular benefits [10, 11]. Sodium-glucose cotransporter-2 inhibitors (SGLT2is), including empagliflozin, dapagliflozin, canagliflozin, and ertugliflozin, show a good prospect in the treatment of HF [12–16]. EMPEROR-Preserved demonstrated the clinical benefit of empagliflozin in patients with HFpEF with or without diabetes [12]. Dapagliflozin significantly improved patient-reported symptoms, physical limitations, and exercise function and was well tolerated in chronic HFpEF [13]. Canagliflozin reduced the risk of cardiovascular death or hospitalized HF [14] and improved Kansas City Cardiomyopathy Questionnaire Total Symptom Score [15] in HFpEF patients. Among patients with diabetes and atherosclerotic cardiovascular disease, another SGLT2i, ertugliflozin was non-inferior to placebo with respect to major adverse cardiovascular events [16]. Based on these high-quality clinical trial results, we can confirm that SGLT2is have a significant therapeutic effect on HFpEF. However, the internal biological mechanisms of SGLT2is for HFpEF are unknown.

Network pharmacology is an interdisciplinary subject, its formation and development mainly benefit from artificial intelligence and big data analysis [17, 18]. The primary advantage of network pharmacology is to emphasize the integrity, systemic and biological network of the research object [19]. With the increasing application of network pharmacology, more and more drugs have been explained at the level of the molecular mechanisms and promoted in clinical practice [19, 20]. In this study, we conducted network pharmacology, which aims to construct a multilevel network through various database searches, high-throughput data analysis, and computer simulations to analyze the relationship of medicines, targets, and diseases [21], to systematically explore the targets of SGLT2is and HFpEF and further excavate the biological pathways and mechanisms of SGLT2is for HFpEF, which may lay a foundation for clinical application and in-depth mechanisms exploration of SGLT2is for HFpEF.

## Material and methods

### Targets screening and networks construction

The chemical structures of SGLT2is, namely empagliflozin, dapagliflozin, canagliflozin, and ertugliflozin, were obtained from PubChem [22] and imported to SwissTargetPrediction [23] for potential targets prediction. Moreover, we used DrugBank [24] and Comparative Toxicogenomics Database [25] to supplement the target information. The HFpEF-associated targets were obtained from DisGeNET [26], GeneCards [27], MalaCards [28], Therapeutic Target Database [29], Comparative Toxicogenomics Database [25], National Center for Biotechnology Information, DrugBank [24], and Online Mendelian Inheritance in Man (OMIM). All targets were transformed in the UniProt database [30]. We set up the drug-target and drug-target-disease networks successively using Cytoscape [31]. Additionally, we conducted CytoNCA plugin to calculate and evaluate analysis for several centralities of the unweighted network [32].

### Functional analysis

We used Metascape [33] for gene annotation, pathway and process enrichment analysis, and protein–protein interaction enrichment analysis. For each given gene list, pathway and process enrichment analysis was carried out with the following ontology sources: Gene Ontology (GO) molecular functions, GO cellular components, GO biological processes, Kyoto Encyclopedia of Genes and Genomes (KEGG) Pathway, Reactome Gene Sets, and Pattern Gene Database (PaGenBase). Finally, KEGG Mapper was used to mapper specific signaling pathways [34]. Protein–protein interaction (PPI) enrichment analysis was carried out and the Molecular Complex Detection (MCODE) algorithm was applied to identify densely connected network components [35]. Finally, PaGenBase was conducted to analyze the cell and tissue specificity [36].

### Computational validation

We conducted the receptor-ligand molecular docking to assess these interactions [37]. We chose these targets shared by all four SGLT2is and then obtained their structures from Protein Data Bank [38]. AutoDock Vina [39], PyMOL Molecular Graphics System [40], and Discovery Studio were utilized for molecular docking.

### Statistical analysis

For pathway and process enrichment, the minimum overlap was set to 3, *P*-value was set to 0.01, and the minimum enrichment was set to 1.5. For PPI enrichment, physical core database was chosen and the minimum and the maximum network sizes were set to 3 and 500, respectively.

## Results

### Screening targets of SGLT2is and HFpEF

We firstly obtained the chemical structures of empagliflozin, dapagliflozin, canagliflozin, and ertugliflozin from PubChem (Table 1, Additional file 1: Figure S1). Through various databases, we got 112, 147, 114, and 114 targets in empagliflozin, dapagliflozin, canagliflozin, and ertugliflozin, respectively. We obtained 1505 HFpEF related targets.

**Table 1:** Details of four SGLT2is

SGLT2is	PubChem CID	Molecular Formula	Molecular Weight	Modify Data
Empagliflozin	11,949,646	C_23_H_27_ClO_7_	450.9	2021-07-17
Canagliflozin	24,812,758	C_24_H_25_FO_5_S	444.5	2021-07-17
Dapagliflozin	9,887,712	C_21_H_25_ClO_6_	408.9	2021-07-17
Ertugliflozin	44,814,423	C_22_H_25_ClO_7_	436.9	2021-07-17

### Network construction

**Fig. 1:**
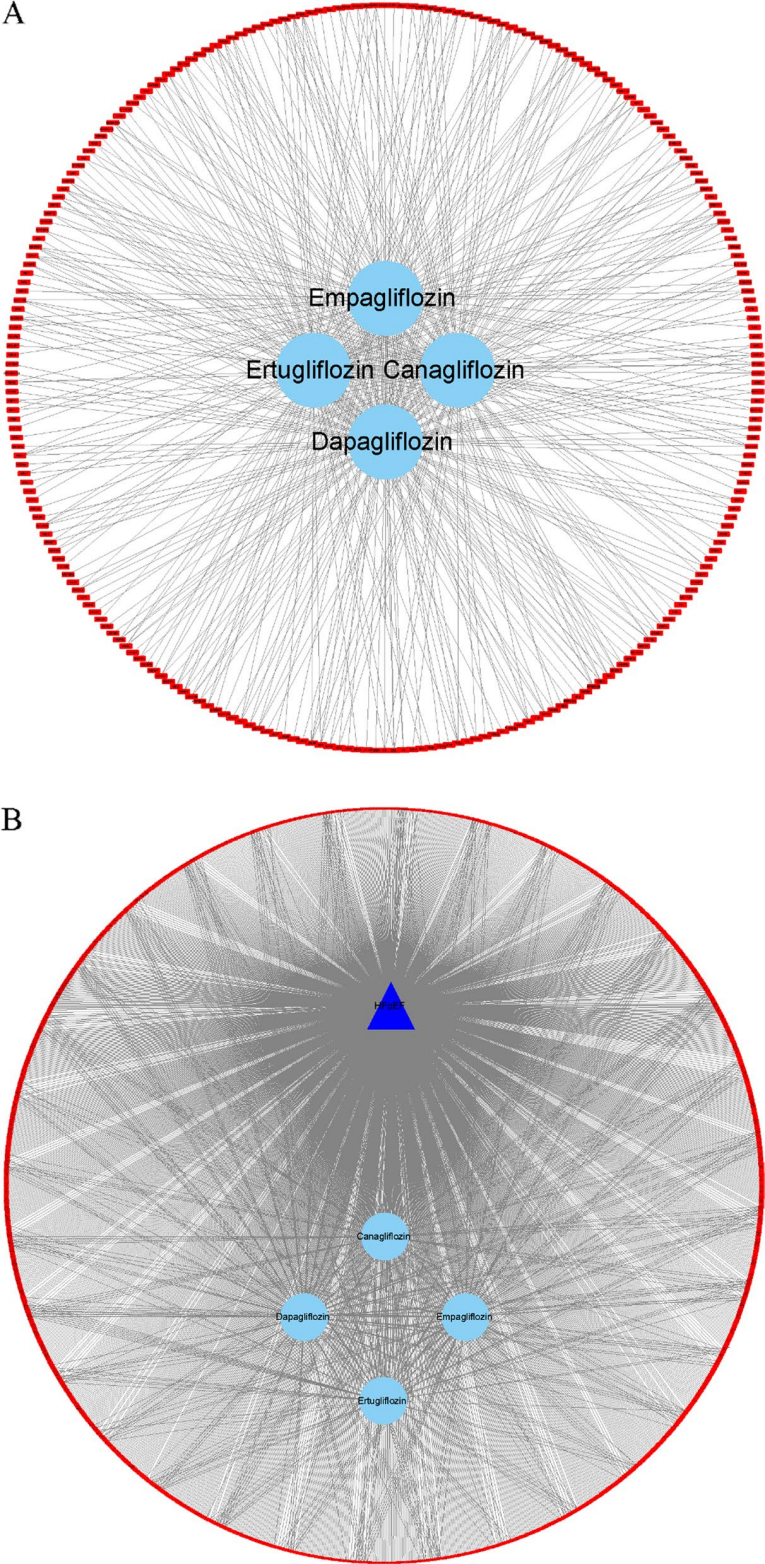
Network construction. **A** The drug-target network. **B** The drug-target-disease network. The red rectangle nodes represent targets, sky blue ellipse nodes represent drugs, namely four SGLT2is (empagliflozin, dapagliflozin, canagliflozin, and ertugliflozin), and the navy blue triangle nodes represent disease, namely HFpEF. The edges mean that nodes can interact with each other

Then we constructed the drug-target network, which was composed of 258 nodes (4 SGLT2is, and 254 targets) and 479 edges (Fig. 1A). Then the drug-target-disease network, which is composed of 1647 nodes (1 HFpEF, 4 SGLT2is, and 1642 targets) and 1984 edges, was built (Fig. 1B). After evaluating by CytoNCA, we identified these targets shared by HFpEF and all SGLT2is (degree = 5), and the details are shown in Table 2.

**Table 2:** Multiple centrality measures in the drug-target-disease network (top 21)

Nodes	Subgragh	Degree	Eigenvector	Information	Closeness	Betweenness
HFpEF	4.02E + 16	1505	0.703902	2.108198	0.855509	2,654,751
Dapagliflozin	1.53E + 14	145	0.043505	2.081154	0.354436	142,937.1
Canagliflozin	8.19E + 13	116	0.031785	2.073808	0.350064	106,420.2
Empagliflozin	6.63E + 13	111	0.028588	2.072172	0.349321	115,219.2
Ertugliflozin	6.50E + 13	107	0.028322	2.070733	0.348729	85,940.67
SLC5A2	3.71E + 13	5	0.021382	1.562157	0.500761	7363.079
SLC5A1	3.71E + 13	5	0.021382	1.562157	0.500761	7363.079
ADK	3.71E + 13	5	0.021382	1.562157	0.500761	7363.079
PDGFRB	3.71E + 13	5	0.021382	1.562157	0.500761	7363.079
ADORA2A	3.71E + 13	5	0.021382	1.562157	0.500761	7363.079
GBA	3.71E + 13	5	0.021382	1.562157	0.500761	7363.079
PDE5A	3.71E + 13	5	0.021382	1.562157	0.500761	7363.079
F3	3.71E + 13	5	0.021382	1.562157	0.500761	7363.079
CTSL	3.71E + 13	5	0.021382	1.562157	0.500761	7363.079
EGFR	3.71E + 13	5	0.021382	1.562157	0.500761	7363.079
GAPDH	3.71E + 13	5	0.021382	1.562157	0.500761	7363.079
MMP3	3.71E + 13	5	0.021382	1.562157	0.500761	7363.079
MMP1	3.71E + 13	5	0.021382	1.562157	0.500761	7363.079
P2RY12	3.71E + 13	5	0.021382	1.562157	0.500761	7363.079
JAK2	3.71E + 13	5	0.021382	1.562157	0.500761	7363.079
MME	3.71E + 13	5	0.021382	1.562157	0.500761	7363.079
MAPK1	3.71E + 13	5	0.021382	1.562157	0.500761	7363.079
ECE1	3.71E + 13	5	0.021382	1.562157	0.500761	7363.079
CDK2	3.71E + 13	5	0.021382	1.562157	0.500761	7363.079
MGAM	3.71E + 13	5	0.021382	1.562157	0.500761	7363.079
SI	3.71E + 13	5	0.021382	1.562157	0.500761	7363.079

### Functional enrichment analysis

**Fig. 2:**
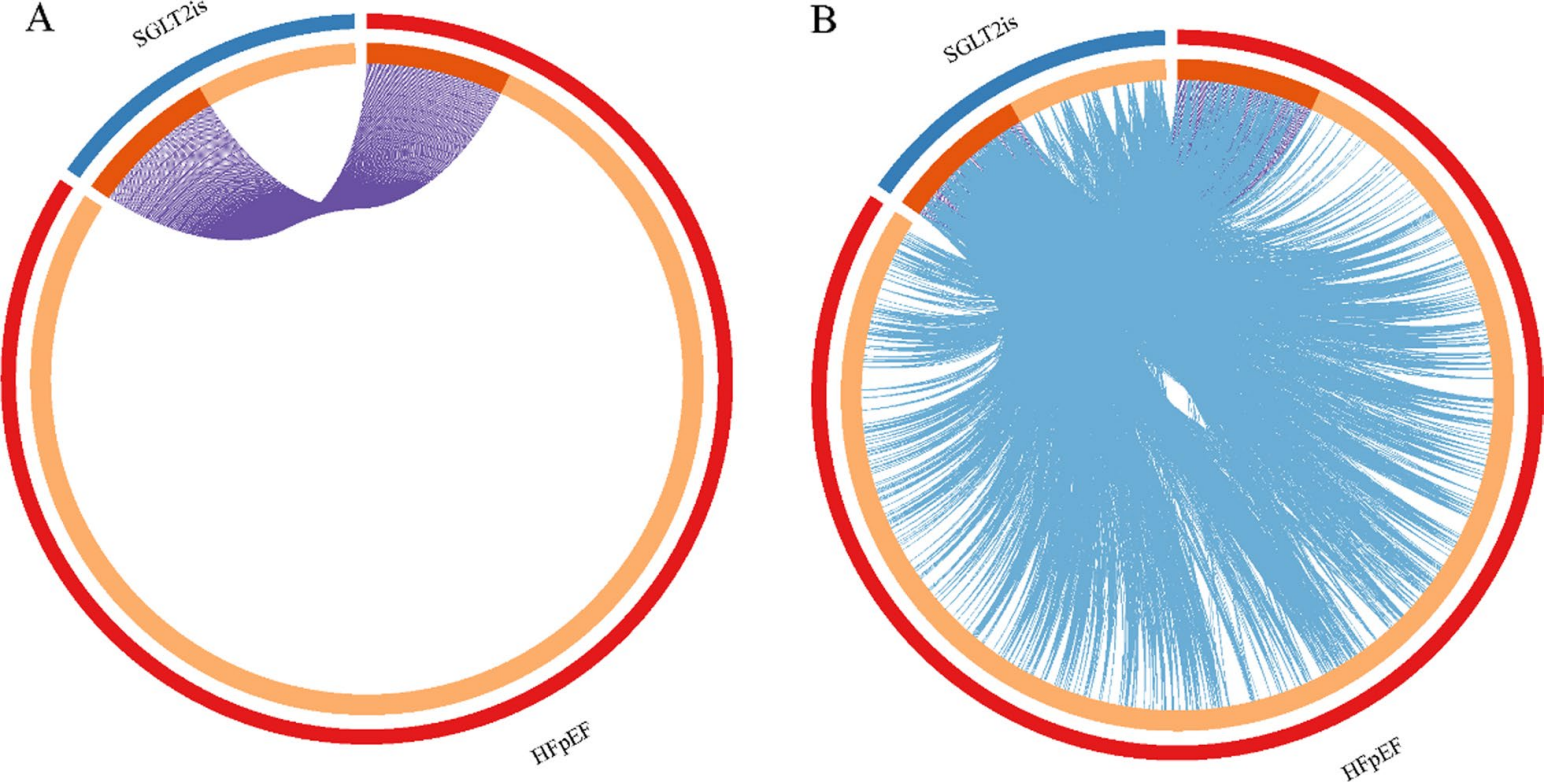
Overlap targets between HFpEF and SGLT2is. **A** Only at the gene level, where purple curves link identical genes. **B** Including the shared term level, where blue curves link genes that belong to the same enriched ontology term. The inner-circle represents gene lists, where hits are arranged along the arc. Genes that hit multiple lists are colored in dark orange, and genes unique to a list are shown in light orange

**Fig. 3:**
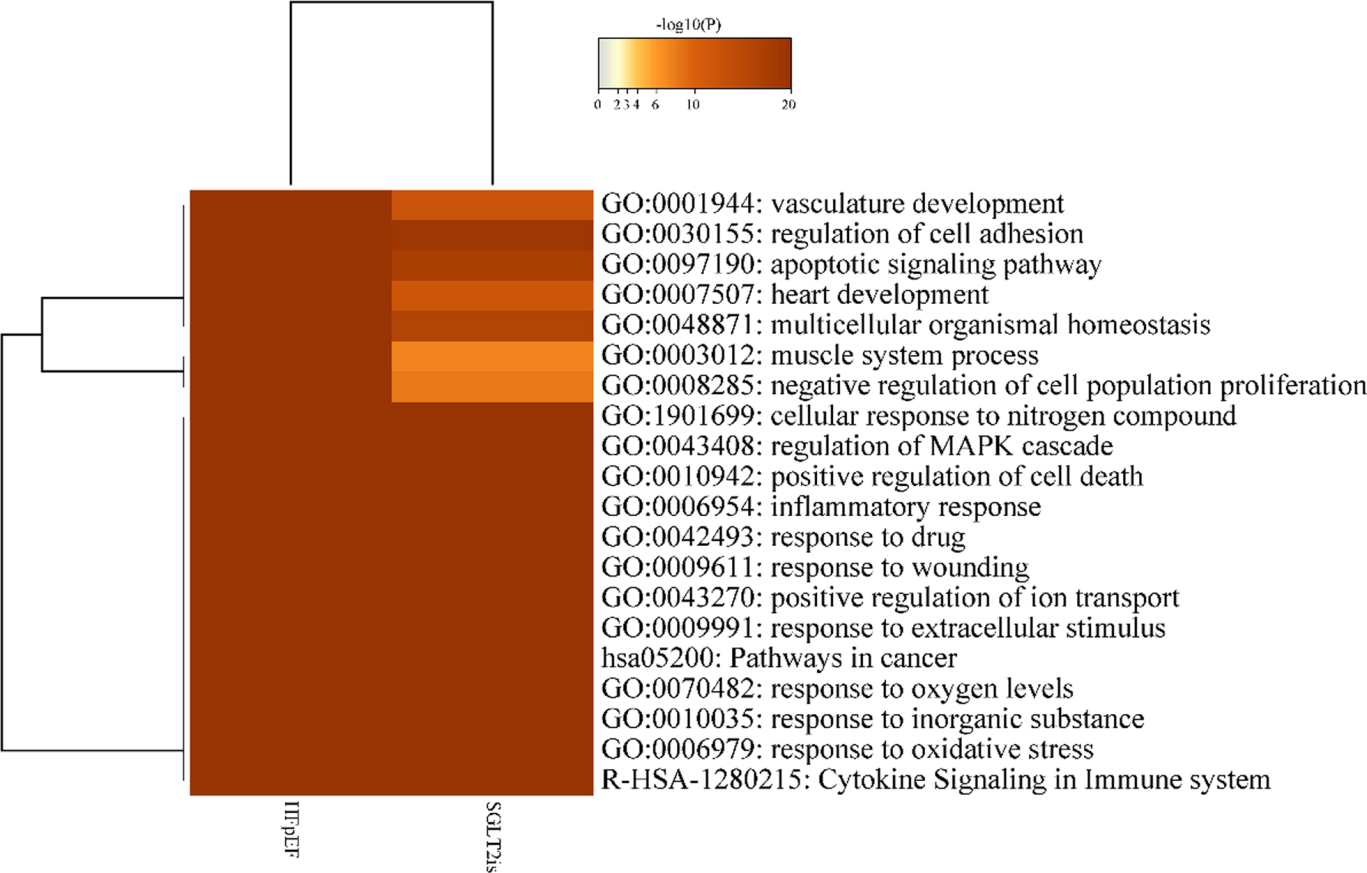
Heatmap of enriched terms across targets, colored by *P*-values (top 20). *P*-values were calculated based on the accumulative hypergeometric distribution

The overlaps between these targets associated with SGLT2is and HFpEF are shown in a Circos plot (Fig. 2A). Another useful representation was to overlap targets based on their functions or shared pathways. The overlaps between targets can be significantly improved by considering overlaps between genes sharing the same enriched ontology terms (Fig. 2B). From the top 20 heatmap of enriched terms across all targets (Fig. 3), we revealed that various ontology terms related to the cardiac and cardiovascular systems, such as vasculature development, response to oxidative stress, inflammatory response, positive regulation of cell death, regulation of MAPK cascade, cellular response to nitrogen compound, negative regulation of cell population proliferation, muscle system process, heart development, apoptotic signaling pathway, and regulation of cell adhesion. Up to 100 enriched clusters (Figure S2A), we viewed apoptosis, cAMP signaling pathway, regulation of lipid metabolic process, heart morphogenesis, and myofibril.

**Fig. 4:**
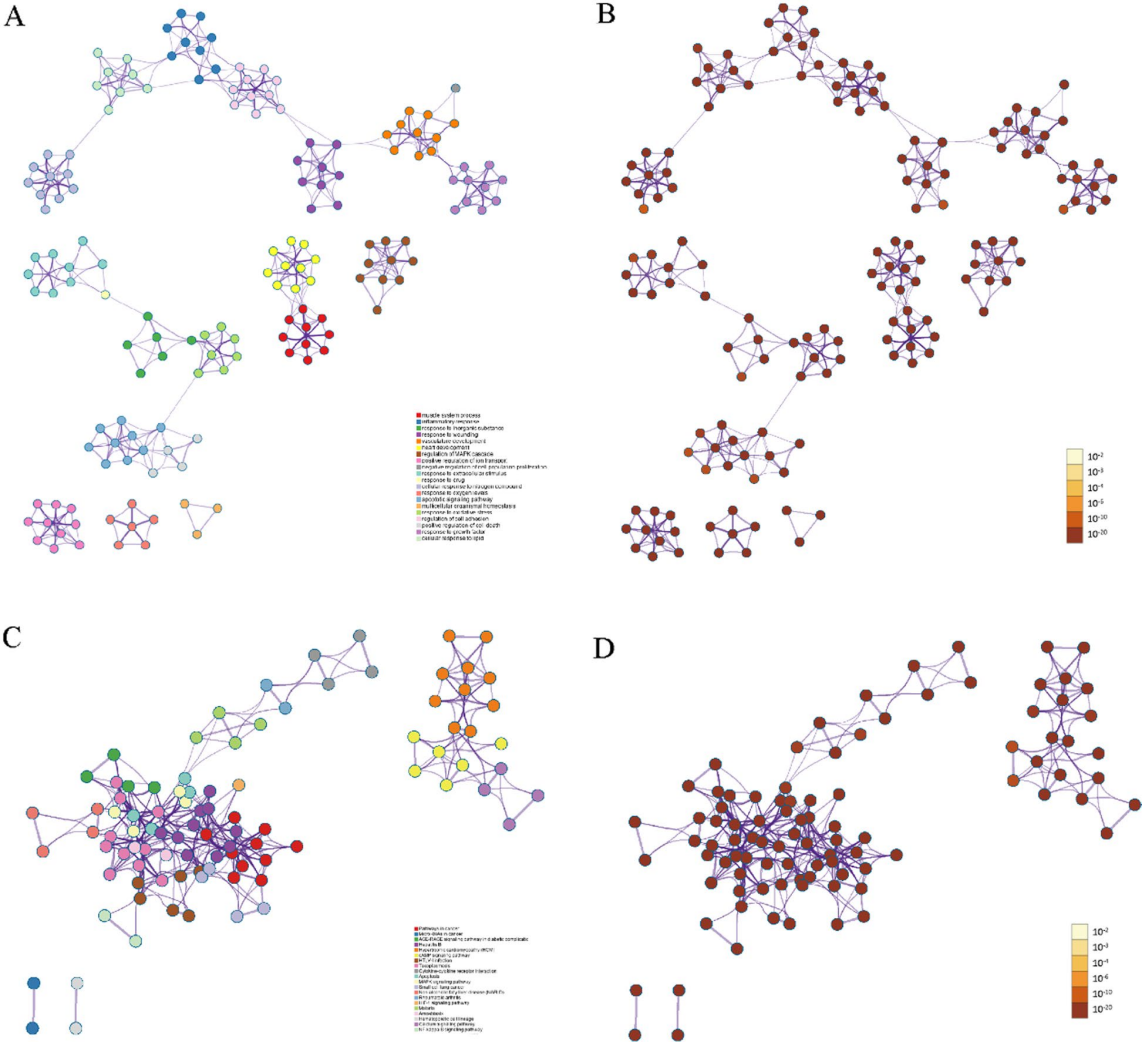
Functional enrichment analysis. **A** GO analysis colored by cluster, where nodes that share the same cluster are typically close to each other. **B** GO analysis colored by *P*-value, where terms containing more genes tend to have a more significant *P*-value. **C** KEGG pathway enrichment analysis colored by cluster, where nodes that share the same cluster are typically close to each other. **D** KEGG pathway enrichment analysis colored by *P*-value, where terms containing more genes tend to have a more significant *P*-value. Only the top 20 results by *P*-value were shown. *P*-values were calculated based on the accumulative hypergeometric distribution

**Fig. 5:**
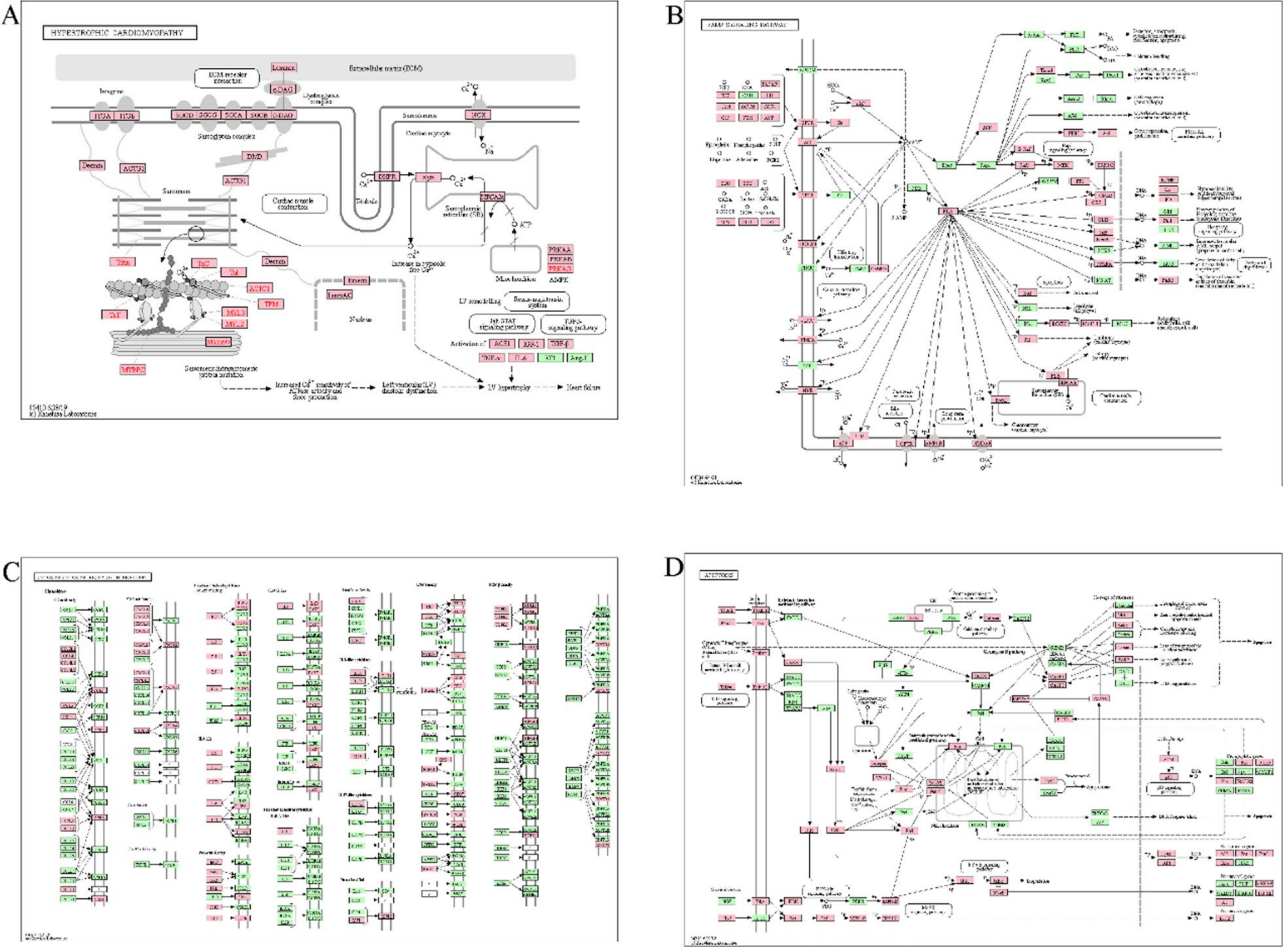
KEGG pathway maps. **A** Hypertrophic cardiomyopathy. **B** cAMP signaling pathway. **C** Cytokine-cytokine receptor interaction. **D** Apoptosis. The pink pentagrams indicate the targets

According to the GO analysis, the top 20 results were all attributed to biological processes (Table 3, Fig. 4A&B). Detailly, the top 10 results of GO molecular functions, GO cellular components, GO biological processes, and KEGG are shown in Figure S2B. Similarly, KEGG results enriched for many pathways known to be associated with cardiovascular disease (Table 4, Fig. 4C&D), such as cardiomyopathy (Fig. 5A), cAMP signaling pathway (Fig. 5B), cytokine-cytokine receptor interaction (Fig. 5C), apoptosis (Fig. 5D), MAPK signaling pathway (Figure S3A), HIF-1 signaling pathway (Figure S3B), calcium signaling pathway (Figure S3C), and NF-kappa B signaling pathway (Figure S3D). In addition, we represented the nodes in GO and KEGG networks as pie charts to particularly visualize whether the terms were shared by SGLT2is and HFpEF or unique SGLT2is or HFpEF, as well understand how these terms are associated with each other within the biological context of the meta study (Figure S4).

**Table 3:** GO clusters with their representative enriched terms (top 20)

GO	Category	Description	Count	%	Log10(P)	Log10(Q)
GO:0,003,012	GO Biological Processes	Muscle system process	192	12.13	−100.00	−96.35
GO:0,006,954	GO Biological Processes	Inflammatory response	240	15.16	−100.00	−96.35
GO:0,010,035	GO Biological Processes	Response to inorganic substance	188	11.88	−97.16	−93.59
GO:0,009,611	GO Biological Processes	Response to wounding	202	12.76	−93.36	−89.85
GO:0,001,944	GO Biological Processes	Vasculature development	217	13.71	−90.37	−86.98
GO:0,007,507	GO Biological Processes	Heart development	183	11.56	−90.06	−86.71
GO:0,043,408	GO Biological Processes	Regulation of mapk cascade	206	13.01	−89.89	−86.58
GO:0,043,270	GO Biological Processes	Positive regulation of ion transport	196	12.38	−87.61	−84.34
GO:0,008,285	GO Biological Processes	Negative regulation of cell population proliferation	198	13.66	−85.08	−81.90
GO:0,009,991	GO Biological Processes	Response to extracellular stimulus	164	10.36	−83.79	−80.72
GO:0,042,493	GO Biological Processes	Response to drug	141	8.91	−80.81	−77.84
GO:1,901,699	GO Biological Processes	Cellular response to nitrogen compound	193	12.19	−79.67	−76.75
GO:0,070,482	GO Biological Processes	Response to oxygen levels	144	9.10	−79.13	−76.26
GO:0,097,190	GO Biological Processes	Apoptotic signaling pathway	173	10.93	−77.38	−74.53
GO:0,048,871	GO Biological Processes	Multicellular organismal homeostasis	162	10.23	−76.02	−73.21
GO:0,006,979	GO Biological Processes	Response to oxidative stress	149	9.41	−73.85	−71.12
GO:0,030,155	GO Biological Processes	Regulation of cell adhesion	189	11.94	−72.88	−70.18
GO:0,010,942	GO Biological Processes	Positive regulation of cell death	178	11.24	−72.87	−70.18
GO:0,070,848	GO Biological Processes	Response to growth factor	189	11.94	−72.67	−69.98
GO:0,071,396	GO Biological Processes	Cellular response to lipid	156	10.76	−70.88	−68.11

"Count" is the number of genes in the gene list with membership in the given ontology term. "%" is the percentage of all of the genes that are found in the given ontology term (only input genes with at least one ontology term annotation are included in the calculation). "Log10(P)" is the *P*-value in log base 10. "Log10(Q)" is the multi-test adjusted *P*-value in log base 10

**Table 4:** KEGG clusters with their representative enriched terms (top 20)

GO	Category	Description	Count	%	Log10(P)	Log10(Q)
hsa05200	KEGG Pathway	Pathways in cancer	150	9.48	−83.58	−80.68
ko05206	KEGG Pathway	MicroRNAs in cancer	109	6.89	−58.39	−56.10
ko04933	KEGG Pathway	AGE-RAGE signaling pathway in diabetic complications	65	4.11	−56.03	−53.91
hsa05161	KEGG Pathway	Hepatitis B	78	4.93	−49.13	−47.31
hsa05410	KEGG Pathway	Hypertrophic cardiomyopathy (HCM)	54	3.72	−48.32	−46.54
hsa04024	KEGG Pathway	cAMP signaling pathway	84	5.31	−45.78	−44.09
hsa05166	KEGG Pathway	HTLV-I infection	85	5.37	−41.85	−40.27
hsa05145	KEGG Pathway	Toxoplasmosis	58	3.66	−40.54	−39.00
hsa04060	KEGG Pathway	Cytokine-cytokine receptor interaction	81	5.59	−39.00	−37.38
hsa04210	KEGG Pathway	Apoptosis	60	3.79	−37.47	−36.10
hsa04010	KEGG Pathway	MAPK signaling pathway	79	4.99	−36.46	−35.15
hsa05222	KEGG Pathway	Small cell lung cancer	44	2.78	−32.03	−30.82
ko04932	KEGG Pathway	Non-alcoholic fatty liver disease (NAFLD)	57	3.60	−31.99	−30.79
ko05323	KEGG Pathway	Rheumatoid arthritis	44	3.03	−31.95	−30.61
hsa04066	KEGG Pathway	HIF-1 signaling pathway	51	3.22	−31.56	−30.40
ko05144	KEGG Pathway	Malaria	33	2.28	−30.49	−29.27
ko05146	KEGG Pathway	Amoebiasis	44	3.03	−30.42	−29.21
hsa04640	KEGG Pathway	Hematopoietic cell lineage	44	3.03	−30.18	−28.99
hsa04020	KEGG Pathway	Calcium signaling pathway	60	3.79	−29.48	−28.38
ko04064	KEGG Pathway	NF-kappa B signaling pathway	43	2.72	−27.91	−26.90

"Count" is the number of genes in the gene list with membership in the given ontology term. "%" is the percentage of all of the genes that are found in the given ontology term (only input genes with at least one ontology term annotation are included in the calculation). "Log10(P)" is the *P*-value in log base 10. "Log10(Q)" is the multi-test adjusted *P*-value in log base 10

**Fig. 6:**
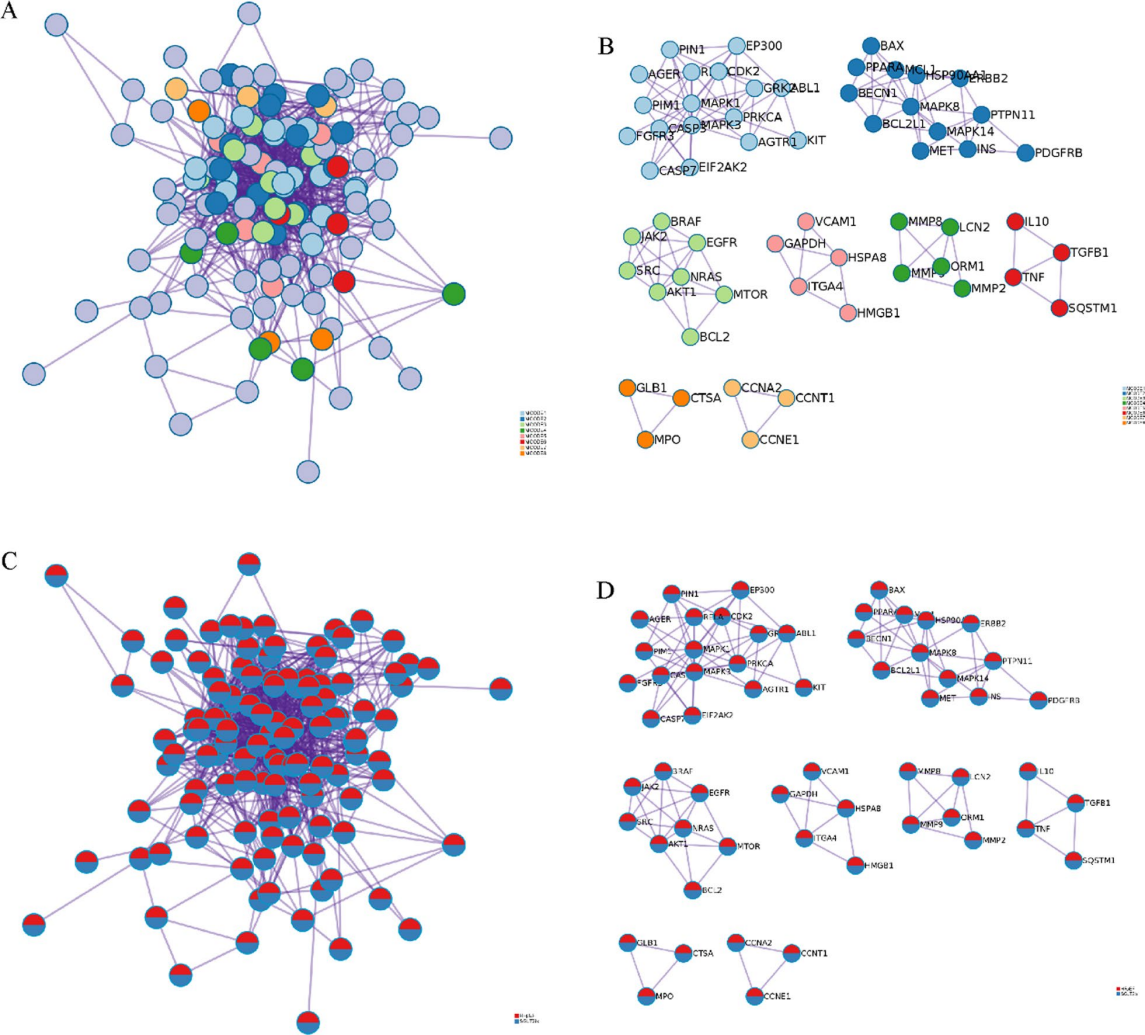
PPI network and MCODE components. **A** PPI network colored by cluster, where terms containing different colors tend to have different MCODE components. **B** The 8 most significant MCODE components form the PPI network colored by cluster. **C** PPI network colored by counts, where terms containing different colors tend to have different MCODE components. **D** The 8 most significant MCODE components form the PPI network colored by counts

Through the PPI network, we demonstrated that targets in HFpEF were enriched to blood circulation, muscle system process, and circulatory system process (Table S1), whereas targets in SGLT2is were enriched to phosphotransferase activity, kinase activity, and protein kinase activity (Figure S5A, Table S1). A total of 11 MCODE components were enriched (Figure S5B, Table S1). Through all targets, PPI network, response to reactive oxygen species, cellular response to oxidative stress, and cellular response to chemical stress were top 3 pathway and process enrichment results (Fig. 6A&C, Table S1). A total of 8 MCODE components were retained (Fig. 6B&D, Table S1). In a word, these enrichment findings support the potential pharmacological mechanisms of SGLT2is for HFpEF.

The enrichment analysis in PaGenBase demonstrated that these targets were specific in smooth muscle, heart, and blood tissue and adipocyte, human umbilical vein endothelial cells, cardiac myocytes, THY^+^, CD33^+^ myeloid cells (Figure S6, Table S2).

### Computational validation

**Fig. 7:**
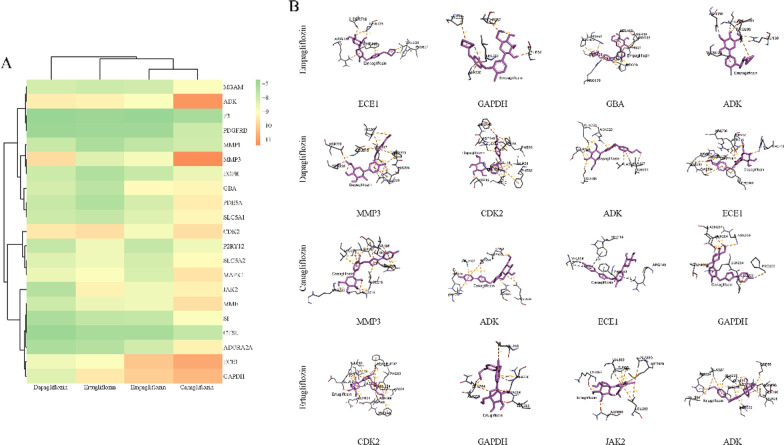
Molecular docking. **A** Heatmap of the molecular docking of 4 SGLT2is with 21 core targets. The color represents the binding energy. The oranger the color, the lower is the binding energy, and the higher is the affinity between the receptor and ligand. **B** Local docking mode between 4 SGLT2is and 4 key targets with the lowest binding energies

In the present study, the binding energies of four SGLT2is and 21 targets were all below -5.0 kcal/mol (Fig. 7A), indicating that these ligands and receptors could bind stability and spontaneously [41]. The local and whole docking mode between four SGLT2is and 4 key targets with the lowest binding energies are shown in Fig. 7B and Figure S7, respectively.

## Discussion

With the release of PRESERVED-HF [13], CANVAS [14], CHIEF-HF [15], and VERTIS CV [16], SGLT2is seem a potential promising therapy for patients with HFpEF. In addition to the recently published EMPEROR-Preserved trial [12], the efficacy of SGLT2is in HFpEF is investigated in 2 additional still ongoing phase 3 trials: DELIVER [42] and CANONICAL [43]. The exact underlying mechanisms of action of SGLT2is in HFpEF have not yet been elucidated. Here, we identified the systemic mechanisms of SGLT2is in the treatment of HFpEF, and our main findings can be summarized as follows: (I) we firstly obtain 487 SGLT2is related targets and 1505 HFpEF related targets. Among SGLT2is related targets, we found there were 21 overlapping targets, which means that these 21 targets can act on four different drugs at the same time; (II) functional enrichment analysis revealed that the targets from SGLT2is were involved in various HFpEF-associated biological processes, such as muscle system process, inflammatory response, response to inorganic substance, response to wounding, vasculature development, heart development, regulation of MAPK cascade, positive regulation of ion transport, negative regulation of cell population proliferation, response to extracellular stimulus, response to drug, cellular response to nitrogen compound, response to oxygen levels, apoptotic signaling pathway, multicellular organismal homeostasis, response to oxidative stress, regulation of cell adhesion, positive regulation of cell death, response to growth factor, and cellular response to lipid; (III) KEGG results were related to hypertrophic cardiomyopathy, cAMP signaling pathway, cytokine-cytokine receptor interaction, apoptosis, MAPK signaling pathway, HIF-1 signaling pathway, calcium signaling pathway, and NF-kappa B signaling pathway, which are associated with HF.

In the present study, we included four SGLT2is, namely empagliflozin, dapagliflozin, canagliflozin, and ertugliflozin. We predicted the targets of SGLT2is in SwissTargetPrediction [23] and supplemented the targets from other databases. Similarly, we used as many databases as possible to collect targets of HFpEF to avoid missing important targets. Moreover, all targets were transformed in the UniProt database [30], which avoids the confusion caused by the aliases of the targets. Here, we identified 21 potential targets, which seem to provide references for SGLT2is in the treatment of HFpEF. SGLT1 and SGLT2, encoded by SLC5A1 and SLC5A2 respectively, are important mediators of epithelial glucose transport, and together with SI are SGLT2is targets [44]. Cardiomyocyte PDGFRB is a regulator of the compensatory cardiac response to pressure overload-induced stress [45]. MMP 3 was associated with focal fibrosis and diffuse fibrosis in HFpEF [46]. Patients and dogs with HF have increased expression of MMP1 [47, 48], suggesting progressive left ventricular remodeling. Selective PDE5A inhibition rescues left ventricular dysfunction, inflammatory immune response, and cardiac remodeling in HF [49, 50]. Studies have proved that P2RY12, MME, ADORA2A, MAPK1, EGFR, and ECE1 were potential targets for the treatment of HFpEF [51–56]. ADK inhibition augments microvascular dilator function and conducted vasodilation and prevents left ventricle diastolic dysfunction in HFpEF [57, 58], CTSL is critical for cardiac morphology and function [59]. JAK2/STAT3 pathway [60, 61], EGFR/Akt/ERK1/2 axis [62], and p27/CDK2/mTOR axis [63] linked to HF.

Different multidirectional mechanisms of SGLT2is could improve HF status [64]. However, there are few known mechanisms of SGLT2is in HFpEF. A previous study indicated that SGLT2is may upregulate the renin–angiotensin–aldosterone system [65]. Empagliflozin could improve diastolic stiffness, hence diastolic function [66], attenuate cardiac fibrosis, and improve ventricular hemodynamics [67]. Empagliflozin beneficially reduced myofilament passive stiffness by enhancing phosphorylation levels of myofilament regulatory proteins in myocardial fibers from patients and rats with HFpEF [66]. We also found that cell proliferation, apoptosis, as well as organismal homeostasis were important in the biological processes of HFpEF from the present study, which are consistent with available evidence [68–70]. Moreover, we highlighted vasculature development, heart development, and ion transport in SGLT2is treatment of HFpEF, which are also consistent with the current cognition [52, 53]. Empagliflozin reduced the activity of the cardiac Na^+^/H^+^ exchanger to possibly improve cardiac function [71, 72]. Later, it was found that dapagliflozin and canagliflozin inhibited Na^+^/H^+^ exchanger activity and reduced cytosolic Na^+^ [73]. Additionally, empagliflozin reduced Ca^2+^/calmodulin-dependent kinase II (CaMKII) activity and CaMKII-dependent sarcoplasmic reticulum Ca^2+^ leak [74, 75].

A large number of studies have shown that HFpEF is a syndrome of over-activation of inflammatory, oxidative stress, and autophagy [76], which is also consistent with our results. Dapagliflozin decreased hypertension and reversed left ventricle concentric remodeling in HFpEF pigs partly by restraining sympathetic tone in the aorta, leading to inhibition of the inflammatory response and NO-cGMP-PKG pathway activation [77]. Empagliflozin reduced inflammatory and oxidative stress in HFpEF and thereby improved the NO-sGC-cGMP-cascade and PKGIα activity via reduced PKGIα oxidation and polymerization [78]. Besides, canagliflozin might exert anti-inflammatory effects by inhibiting intracellular glucose metabolism and promoting autophagy [79].

Through KEGG pathways enrichment analysis, we emphasized the cAMP signaling pathway, MAPK signaling pathway, HIF-1 signaling pathway, calcium signaling pathway, and apoptosis during the occurrence and development of HFpEF. These findings are in line with previous studies. MiR-665 inhibition can stabilize the cardiac function of HF rats via the cAMP signaling pathway via upregulation of the GLP1R [58]. By switching from Gαs to Gαi2 activation, NDPK-C, a novel critical regulator of cAMP signaling and cardiac contractility, may contribute to lower cAMP levels and the related contractile dysfunction in HF [80]. MAPK has been studied in-depth about cardiac development and function [59, 60]. Elucidation of the molecular mechanisms of hypoxia signaling will greatly help us to understand the pathophysiology of cardiovascular disorders [81]. Defective cardiolipin remodeling, upon loss of the cardiolipin acyl transferase tafazzin, decreases HIF-1α signaling in hypoxia [82]. Enhanced activation of the Dyrk1A-ASF-CaMKIIδ signaling pathway may underlie the mechanisms of HF [83]. Numerous drugs can improve HF through the calcium signaling pathway. In addition, PPI network and cell and tissue specificity analysis confirmed the previous results. Importantly, we conducted molecular docking to further verify the interactions and combinations of SGLT2is and core targets. In a word, these enrichment findings support the potential pharmacological mechanisms of SGLT2is for HFpEF.

However, there were some limitations that we should pay attention to. Firstly, when we fished the targets from HFpEF, we found that some databases are not updated to HFpEF, only HF or chronic HF, which makes us inevitably lose some important targets. Moreover, we only generally analyzed the mechanisms of SGLT2is for HFpEF, but we still need to further study the single SGT2i, because the action mechanisms of different SGLT2is may not be completely consistent. In addition, the pathophysiology of HFpEF manifestations is highly heterogeneous [84, 85], more current and future endeavors are underway to evaluate the optimal methods to classify patients into phenotypically homogeneous subpopulations to facilitate better individualization of treatment [86]. Finally, this study is based on computer and biological information mining, these reliable results we obtained here still need to be verified by molecular biology experiments in the later stage.

## Conclusions

In conclusion, we identified the synergistic pharmacological mechanisms of SGLT2is in HFpEF by the integrative virtual screening and network pharmacology method. Moreover, our main findings were validated with molecular docking. Our findings of the potential mechanisms of the direct or indirect synergy of multiple targets and pathways provide an optional therapy for HFpEF. However, more experimental and clinical validation is essential to reveal the effect of SGLT2is against HFpEF.

## Supplementary Information

**Additional file 1**. Tables S1, S2 and Figure S1–S7.

## Data Availability

Chemical structures of SGLT2is were obtained from PubChem (https://pubchem.ncbi.nlm.nih.gov/) with PubChem CID 11,949,646 for empagliflozin, 24,812,758 for canagliflozin, 9,887,712 for dapagliflozin, and 44,814,423 for ertugliflozin.

## Abbreviations

CaMKII: Ca^2+^/calmodulin-dependent kinase II
EF: Ejection fraction
GO: Gene Ontology
HF: Heart failure
HFpEF: Heart failure with preserved ejection fraction
KEGG: Kyoto Encyclopedia of Genes and Genomes
PPI: Protein–protein interaction
SGLT2i: Sodium-glucose cotransporter-2 inhibitor
