# Supplementary material for: Pharmacological mechanisms of sodium-glucose co-transporter 2 inhibitors in heart failure with preserved ejection fraction
Source: BMC Cardiovasc Disord. 2022 Jun 10;22:261. doi: 10.1186/s12872-022-02693-8 (PMC9188076; doi:10.1186/s12872-022-02693-8)
Supplement: Supplementary file 1 — Additional file 1. Tables S1, S2 and Figure S1–S7. [file 12872_2022_2693_MOESM1_ESM.pdf]

Additional files

Table S1. Annotations of the PPI network and MCODE components (top3).

Network	Annotation	Description	Log10(P)
HFpEF	GO:0008015	blood circulation	-100.0
	GO:0003012	muscle system process	-100.0
	GO:0003013	circulatory system process	-100.0
SGLT2is	GO:0016773	phosphotransferase activity, alcohol group as acceptor	-53.4
	GO:0016301	kinase activity	-53.2
	GO:0004672	protein kinase activity	-51.9
SGLT2is_MCODE_ALL	GO:0016773	phosphotransferase activity, alcohol group as acceptor	-36.7
	GO:0016301	kinase activity	-36.4
	GO:0004672	protein kinase activity	-34.3
SGLT2is_SUB1_MCODE_1	GO:0000307	cyclin-dependent protein kinase holoenzyme complex	-16.5
	GO:0045859	regulation of protein kinase activity	-14.6
	GO:1902554	serine/threonine protein kinase complex	-13.8
SGLT2is_SUB1_MCODE_2	GO:0052695	cellular glucuronidation	-19.7
	GO:0032787	monocarboxylic acid metabolic process	-19.7
	GO:0006805	xenobiotic metabolic process	-19.4
SGLT2is_SUB1_MCODE_3	GO:0004672	protein kinase activity	-13.5
	GO:0016773	phosphotransferase activity, alcohol group as acceptor	-12.7
	GO:0019904	protein domain specific binding	-12.6
SGLT2is_SUB1_MCODE_4	GO:0005976	polysaccharide metabolic process	-10.5
	GO:0005977	glycogen metabolic process	-9.3
	GO:0006073	cellular glucan metabolic process	-9.3
SGLT2is_SUB1_MCODE_5	GO:0035578	azurophil granule lumen	-7.1
	GO:0005766	primary lysosome	-6.2
	GO:0042582	azurophil granule	-6.2
SGLT2is_SUB1_MCODE_6	GO:0001609	G protein-coupled adenosine receptor activity	-10.0
	GO:0035588	G protein-coupled purinergic receptor signaling pathway	-8.7
	GO:0001973	G protein-coupled adenosine receptor signaling pathway	-8.7
SGLT2is_SUB1_MCODE_7	GO:0007189	adenylate cyclase-activating G protein-coupled receptor signaling pathway	-5.9
	GO:0007188	adenylate cyclase-modulating G protein-coupled receptor signaling pathway	-5.3

	GO:0001664	G protein-coupled receptor binding	-5.0
SGLT2is_SUB1_MCODE_8	GO:0004558	alpha-1,4-glucosidase activity	-11.4
	GO:0090599	alpha-glucosidase activity	-10.7
	GO:0015926	glucosidase activity	-9.4
SGLT2is_SUB1_MCODE_10	GO:0035580	specific granule lumen	-8.0
	GO:0042581	specific granule	-6.7
	GO:0034774	secretory granule lumen	-5.8
SGLT2is_SUB1_MCODE_11	GO:0006678	glucosylceramide metabolic process	-11.0
	GO:0006677	glycosylceramide metabolic process	-9.7
	GO:0046527	glucosyltransferase activity	-9.5
_FINAL	GO:0000302	response to reactive oxygen species	-29.6
	GO:0034599	cellular response to oxidative stress	-29.2
	GO:0062197	cellular response to chemical stress	-28.9
_FINAL_MCODE_ALL	GO:0000302	response to reactive oxygen species	-25.3
	GO:0062197	cellular response to chemical stress	-25.0
	GO:0051347	positive regulation of transferase activity	-24.7
_FINAL_SUB1_MCODE_1	GO:0004672	protein kinase activity	-12.7
	GO:0016773	phosphotransferase activity, alcohol group as acceptor	-12.0
	GO:1901699	cellular response to nitrogen compound	-11.8
_FINAL_SUB1_MCODE_2	GO:0051896	regulation of protein kinase B signaling	-11.2
	GO:0043491	protein kinase B signaling	-10.9
	GO:0051897	positive regulation of protein kinase B signaling	-10.1
_FINAL_SUB1_MCODE_3	GO:0018105	peptidyl-serine phosphorylation	-10.3
	GO:0032147	activation of protein kinase activity	-10.2
	GO:0018209	peptidyl-serine modification	-10.1
_FINAL_SUB1_MCODE_4	GO:0035987	endodermal cell differentiation	-7.4
	GO:0030574	collagen catabolic process	-7.4
	GO:0001706	endoderm formation	-7.2
_FINAL_SUB1_MCODE_5	GO:0050839	cell adhesion molecule binding	-6.2
	GO:0005178	integrin binding	-5.9
	GO:1903039	positive regulation of leukocyte cell-cell adhesion	-5.3
_FINAL_SUB1_MCODE_6	GO:0002719	negative regulation of cytokine production involved in immune response	-8.6
	GO:0002701	negative regulation of production of molecular mediator of immune response	-8.0
	GO:1902895	positive regulation of pri-miRNA transcription by RNA polymerase II	-8.0
_FINAL_SUB1_MCODE_7	GO:0000307	cyclin-dependent protein kinase holoenzyme complex	-8.5
	GO:0016538	cyclin-dependent protein serine/threonine kinase	-8.3

		regulator activity	
	GO:1902554	serine/threonine protein kinase complex	-7.5
_FINAL_SUB1_MCODE_8	GO:0035578	azurophil granule lumen	-7.5
	GO:0005766	primary lysosome	-6.8
	GO:0042582	azurophil granule	-6.8

Table S2. Summary of enrichment analysis in PaGenBase.

GO	Description	_LogP_HFpEF	_LogP_SGLT2is
PGB:00002	Cell-specific: HEPG2	-13.925	-5.73526
PGB:00049	Cell-specific: Adipocyte	-10.9623	-5.14538
PGB:00001	Tissue-specific: liver	-17.6753	-9.00093
PGB:00004	Tissue-specific: kidney	-4.94603	-7.71121
PGB:00045	Tissue-specific: placenta	-15.5053	-2.17974
PGB:00031	Cell-specific: HUVEC	-11.7767	-2.67481
PGB:00018	Tissue-specific: lung	-12.61	-3.71064
PGB:00078	Cell-specific: liver cell	-9.84714	-2.8477
PGB:00011	Tissue-specific: spleen	-7.37918	-2.68737
PGB:00081	Cell-specific: Bronchial Epithelial Cells	-6.27748	-2.80705
PGB:00015	Tissue-specific: Smooth Muscle	-16.266	0
PGB:00120	Cell-specific: Cardiac Myocytes	-14.1338	0
PGB:00047	Cell-specific: THY^+	-12.6346	0
PGB:00014	Cell-specific: DRG	-10.6015	0
PGB:00037	Tissue-specific: heart	-22.8523	0
PGB:00041	Tissue-specific: Blood	-6.64036	0
PGB:00060	Tissue-specific: retinoblastoma	-7.03765	0
PGB:00069	Cell-specific: A204	-8.6486	0
PGB:00044	Tissue-specific: skeletal muscle	-6.62014	0
PGB:00115	Cell-specific: $CD33^+$ Myeloid	-5.30947	0

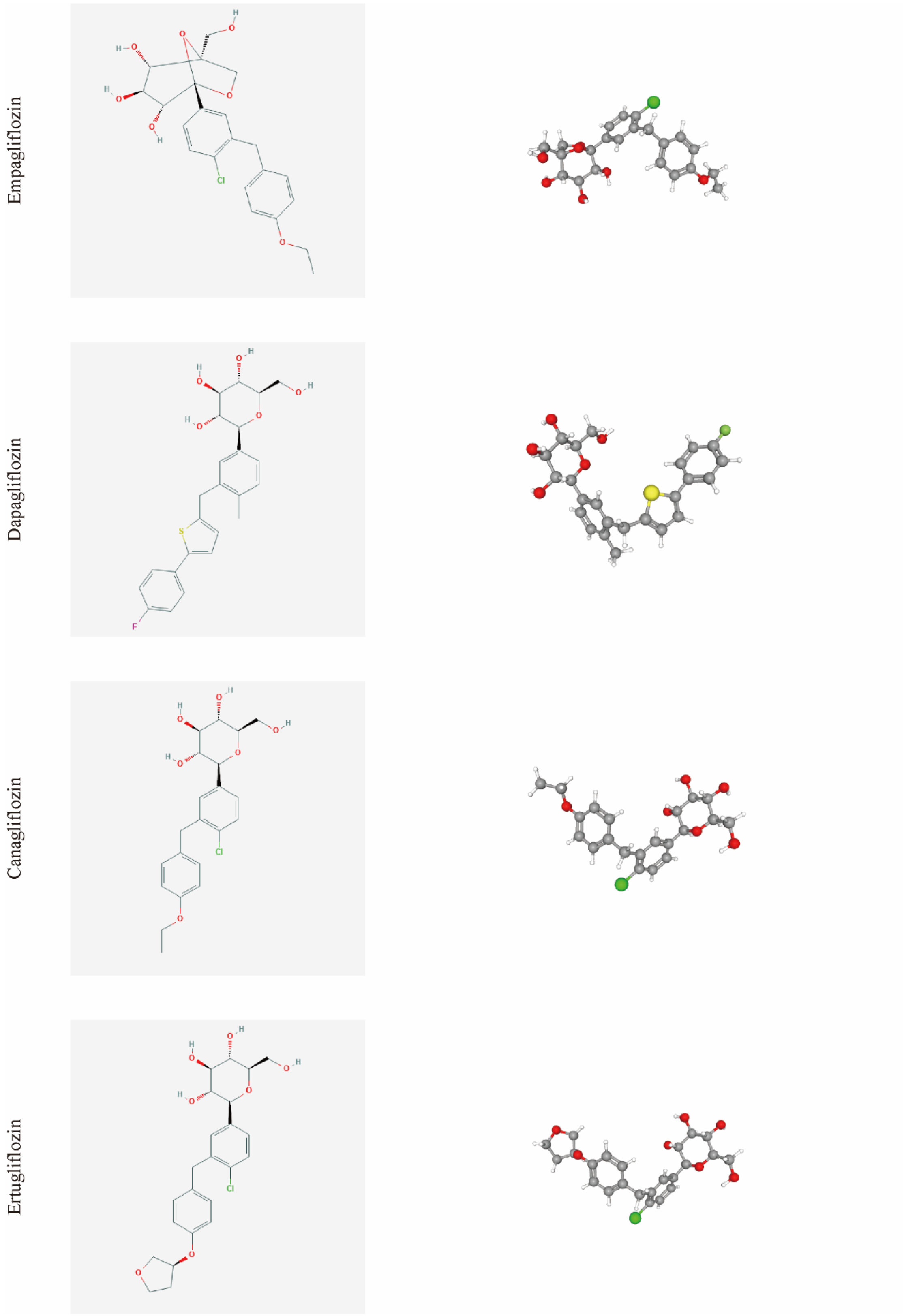

Figure S1. The structures of four SGLT2is.

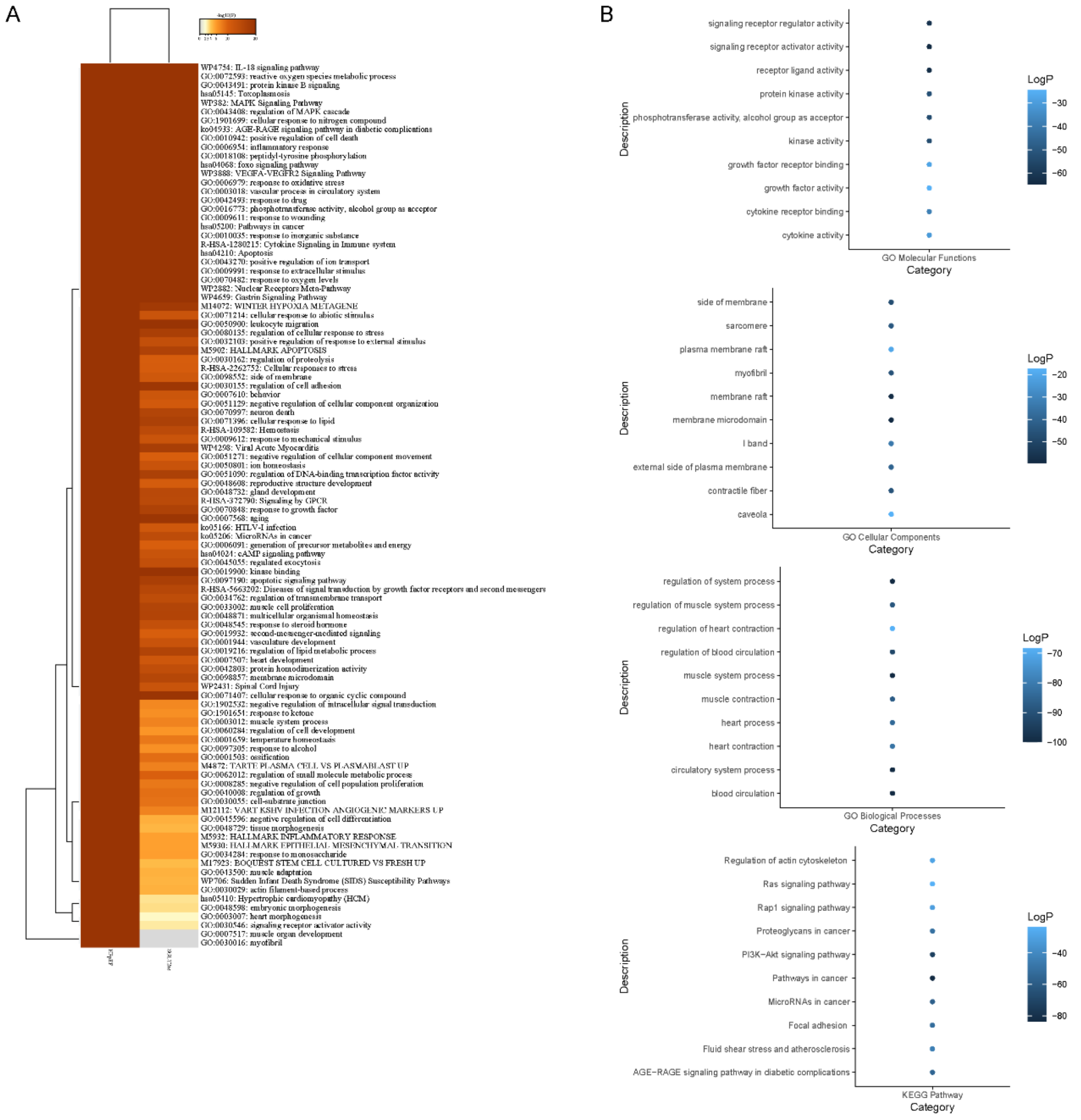

Figure S2. Functional enrichment analysis. A. Heatmap of enriched terms across targets, colored by *P*-values (top 100). *P*-values were calculated based on the accumulative hypergeometric distribution. B. Bubble chart of GO molecular functions, GO cellular components, GO biological processes, and KEGG analysis, colored by *P*-values (top 10).

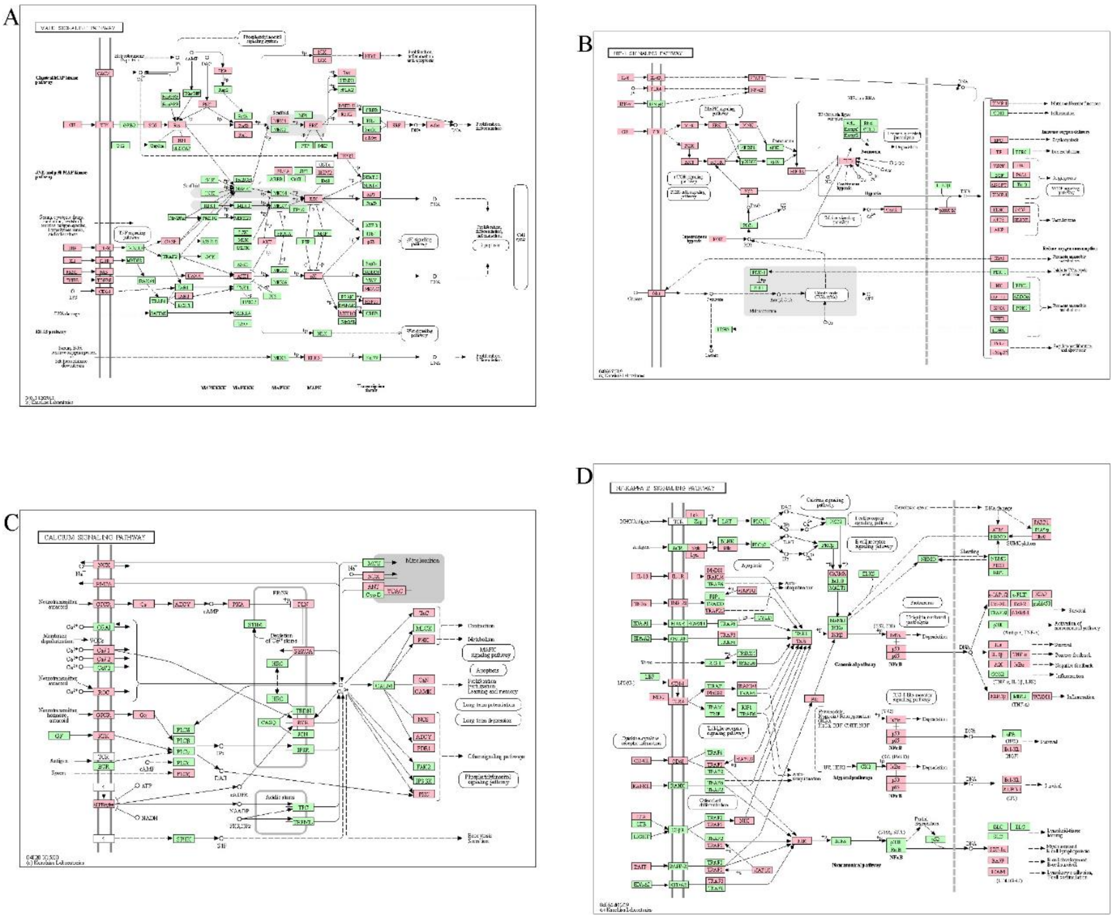

Figure S3. KEGG pathway maps. A. MAPK signaling pathway. B. HIF-1 signaling pathway. C. Calcium signaling pathway. D. NF-kappa B signaling pathway. The pink pentagrams indicate the targets.

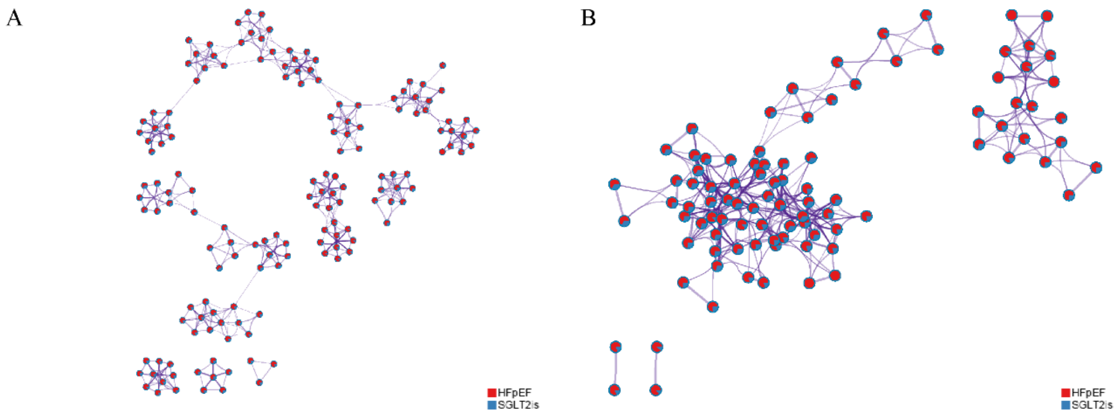

Figure S4. Network of enriched terms represented as pie charts. A. GO. B. KEGG. The pie charts are color-coded based on the gene list identities, where the size of a slice represents the percentage of genes under the term that originated from the corresponding gene list.

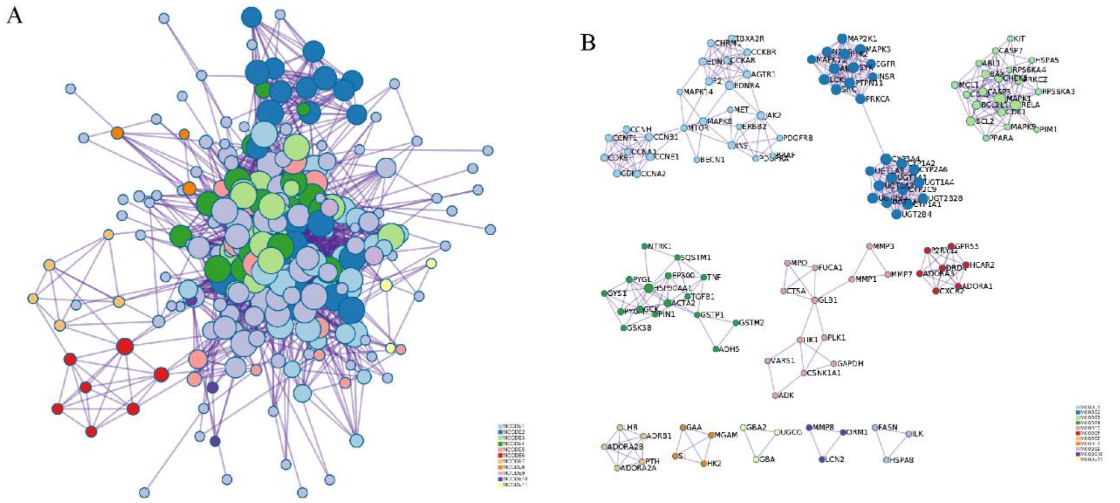

Figure S5. PPI network of SGLT2is and its MCODE components. A. PPI network, where terms containing different colors tend to have different MCODE components. B. The 11 most significant MCODE components form the PPI network.

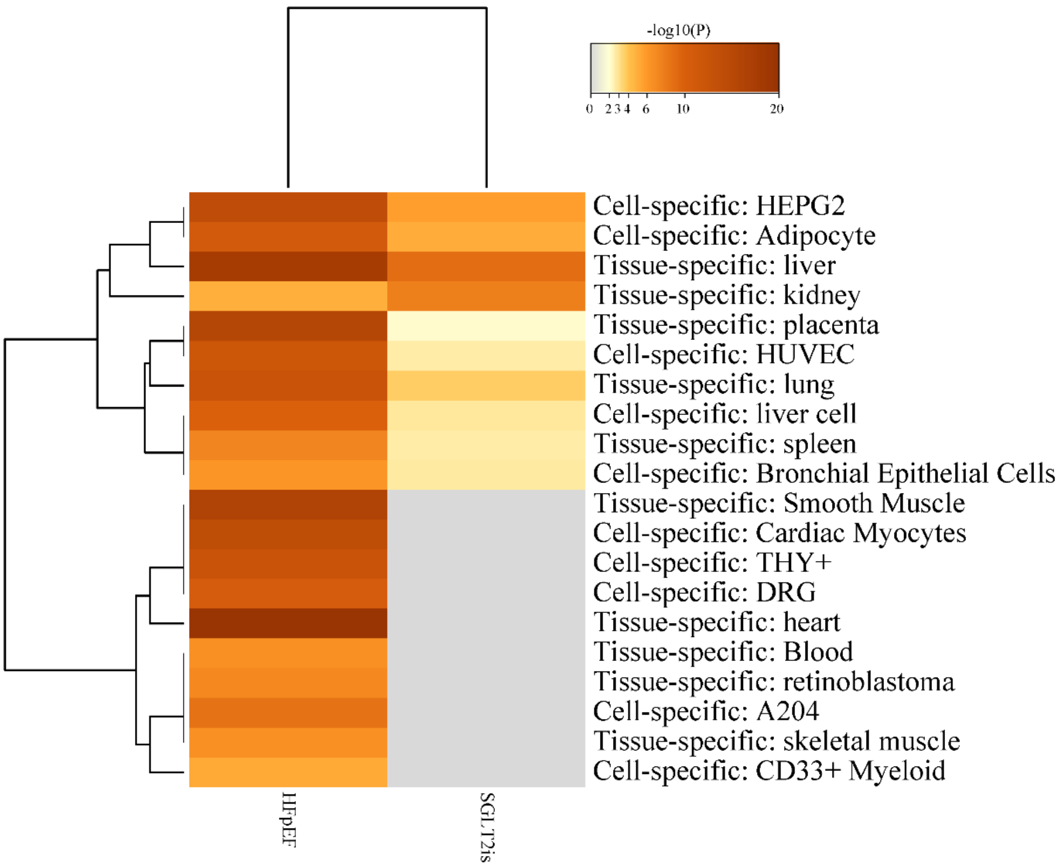

Figure S6. Summary of enrichment analysis in PaGenBase. *P*-values were calculated based on the accumulative hypergeometric distribution.

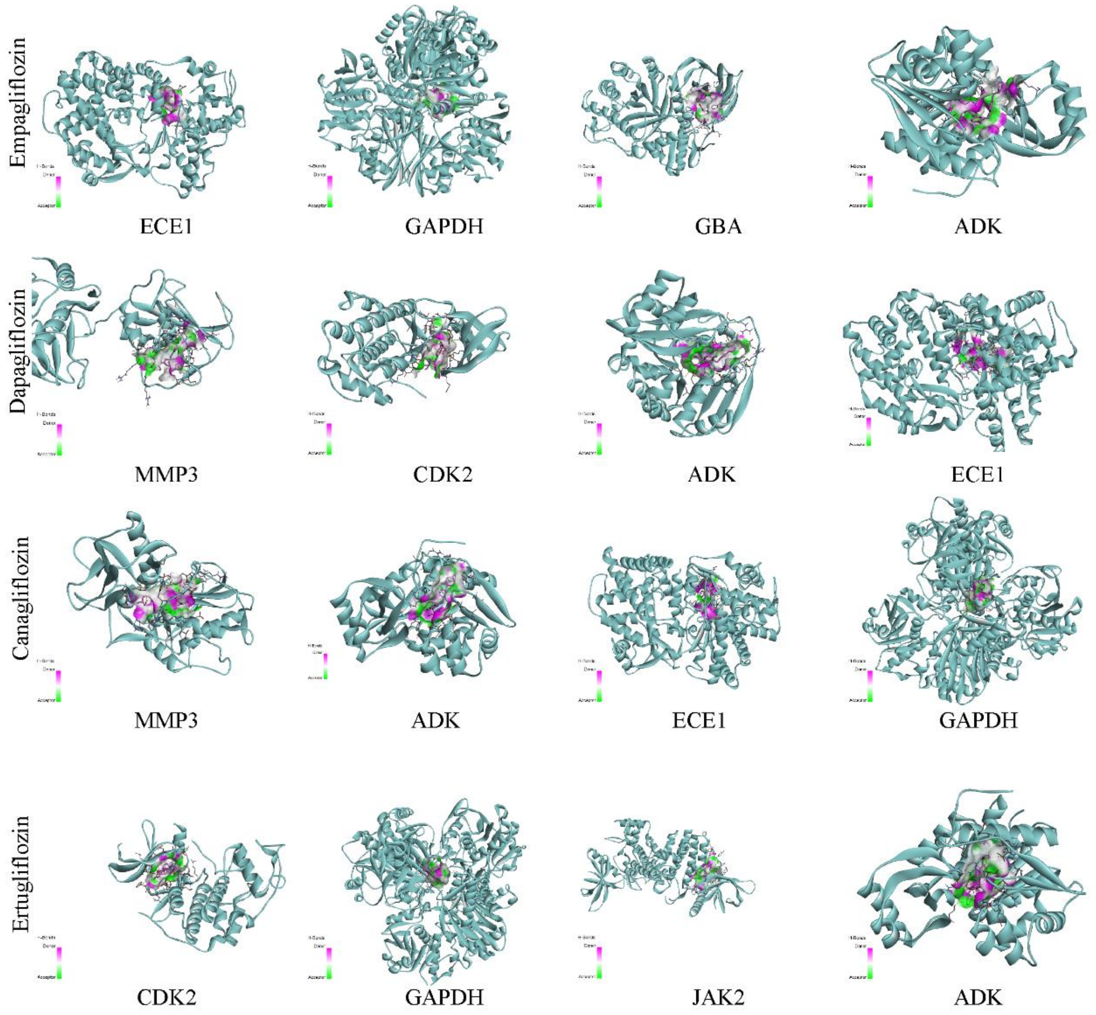

Figure S7. Whole docking mode between SGLT2is and 4 key targets with the lowest binding energies.
